# Psychometric properties of the Korean version of questionnaires on adherence to physical distancing and health beliefs about COVID-19 in the general population

**DOI:** 10.3389/fpsyt.2023.1132169

**Published:** 2023-07-06

**Authors:** Youjin Hong, Hoyoung An, Eulah Cho, Oli Ahmed, Myung Hee Ahn, Soyoung Yoo, Seockhoon Chung

**Affiliations:** ^1^Department of Psychiatry, Gangneung Asan Hospital, University of Ulsan College of Medicine, Gangneung, Republic of Korea; ^2^Department of Psychiatry, Keyo Hospital, Uiwang, Gyeonggi, Republic of Korea; ^3^Department of Psychiatry, Asan Medical Center, University of Ulsan College of Medicine, Seoul, Republic of Korea; ^4^Department of Psychology, University of Chittagong, Chattogram, Bangladesh; ^5^National Centre for Epidemiology and Population Health, Australian National University, Canberra, ACT, Australia; ^6^Division of Psychiatry, Health Screening and Promotion Center, Asan Medical Center, Seoul, Republic of Korea; ^7^Department of Convergence Medicine, University of Ulsan College of Medicine, Seoul, Republic of Korea

**Keywords:** physical distancing, health beliefs, COVID-19, anxiety, depression

## Abstract

**Introduction:**

We aimed to examine the psychometric properties of the Korean version of the questionnaires on adherence to physical distancing and health beliefs about COVID-19 in the general population in South Korea. In addition, we investigated how the various sections interacted with each other and with viral anxiety and depression, and ultimately affected adherence to physical distancing.

**Methods:**

An anonymous online survey was conducted among members of the general population in South Korea between 10 and 18 January 2022. We recruited 400 respondents and measured their demographic information, symptoms, and responses to questions about COVID-19. First, we examined the reliability and validity of the questionnaires, which included questions about people’s adherence to physical distancing guidelines and COVID-19-related health beliefs. Second, we examined the relationship between physical distancing and viral anxiety or depression, as assessed using the six-item Stress and Anxiety to Viral Epidemics (SAVE-6) scale and the Patient Health Questionnaire-9 (PHQ-9).

**Results:**

All 400 participants (204 men, age 41.6 ± 10.8) completed the survey. Confirmatory factor analysis revealed a good model fit for adherence to physical distancing (CFI = 1.000, TLI = 1.019, RMSEA = 0.000, and SRMR = 0.034) and health beliefs about COVID-19 (CFI = 0.993, TLI = 0.991, RMSEA = 0.030, and SRMR = 0.052). It also showed good reliability for Factor I (Cronbach’s α = 0.826) and Factor II (α = 0.740). Four categories of the COVID-19 health beliefs questionnaire also showed good reliability for perceived susceptibility (α = 0.870), perceived severity (α = 0.901), perceived benefit (α = 0.935), and barriers to following physical distancing (α = 0.833). Structural equation models showed that the effects of health beliefs and viral anxiety and depression were mediated mostly by personal injunctive norms. Goodness-of-fit measures indicated a good fit. (Chi-square = 24.425, df = 7, *p* < 0.001; CFI = 0.966; RMSEA = 0.079).

**Conclusion:**

The Korean version of the COVID-19 adherence to physical distancing and health beliefs questionnaires showed good reliability and validity in the Korean general population. In addition, the effects of health beliefs, along with viral anxiety and depression, were mainly mediated by personal injunctive norms.

## Introduction

During the COVID-19 pandemic, physical distancing was a key public policy used to prevent the transmission of the virus, along with hand washing and mask use (1). Adopted by many countries around the world, physical distancing was crucial in reducing the spread of the virus. Its implementation has reduced the risk of contracting COVID-19 from 13 to 25%. Higher levels of adherence (for example, by avoiding public places or gatherings of more than 10 people) can further reduce the risk (2, 3).

Due to its effectiveness, physical distancing has been a critical component of the public response. As a result, many factors that increase adherence have been investigated. Individuals who are female, older, more educated, in a higher socioeconomic group, or non-White are more likely to adopt this protective behavior (4, 5). Similarly, those who trust the government or agency responsible for the policy, hold liberal political views or have fewer pseudoscientific beliefs are more likely to adhere more closely to distancing guidelines (6). Adherence can also be influenced by emotional states and socio-demographic factors. Fear or anxiety related to the virus has been found to be associated with higher compliance with public health guidelines (7), and fostering empathy through outreach to individuals at high risk of viral infection has shown to improve adherence to physical distancing measures (8). Depression severity has been linked to the fear of social distancing (9), while people with better adherence have been shown to have fewer symptoms (10). Personality has also been studied in this context: being agreeable, conscientious, and extraverted may improve adherence (11). Furthermore, reduced social support may reduce adherence (12).

Among numerous previous reports, one study focusing on non-adherent behaviors (13) found that the following factors—vulnerability to COVID-19, an inability to maintain physical distance, pressure from (and the perceptions of) friends and neighbors, and support from friends— influenced those types of behaviors. Because these factors include constructs from the Health Beliefs Model and Social Norms Theory, such approaches may offer an effective way to understand how different factors interact to increase or decrease adherence to social-distancing guidelines.

The theoretical Health Beliefs Model (HBM) was developed to understand and predict the success or failure of health-promoting behaviors (14). Fundamentally, it posits that health-related behavior consists of two components: (1) the desire to avoid being ill, and (2) the belief that certain health-related actions will prevent or cure illness. The model consists of six constructs: perceived susceptibility, perceived severity, perceived benefits, perceived barriers, cues to action, and self-efficacy. The COVID-19 health-belief questionnaire is an evaluation tool based on the Health Beliefs Model (HBM) and is used to measure COVID-19-related health beliefs. It includes five of the original constructs, excluding “cue to action.”

Social norms theory is another promising way to improve our understanding of the factors that influence people to adhere to physical distancing guidelines. This approach analyzes health behavior by focusing on environmental and interpersonal influences, particularly peer perceptions (15). It postulates that peer-influenced perceived norms differ from actual norms. To promote good health behaviors, this gap or misperception must be addressed. The perceived social norms questionnaire used in this study was developed based on the social norms theory (16). It consists of perceived, descriptive, and injunctive social norms. Both injunctive and descriptive social norms both influence the intention to engage in healthy behaviors (17).

During the COVID-19 pandemic, Gouin et al. developed questionnaires on adherence to physical distancing, COVID-19-related health beliefs, and perceived social norms (18). These questionnaires have been used in other studies to predict physical distancing behaviors during the pandemic (19). Although they have been validated by Korean healthcare workers to examine whether viral anxiety mediates the influence of uncertainty intolerance on adherence to physical distancing, no previous study has validated these questionnaires with the general population in South Korea. In this study, we aimed to investigate the psychometric properties of the Korean version of the questionnaires on adherence to physical distancing, health beliefs about COVID-19, and perceived social norms among the general population. Additionally, we examined how the various sections interacted with each other and with viral anxiety and depression, and ultimately affected the adherence to physical distancing.

## Methods

### Participants and procedure

This study was part of a research that examined people’s behavior and attitudes toward physical distancing during the COVID-19 pandemic and its effects on their psychological factors (20). We conducted an anonymous online survey among the general population in South Korea through the professional survey company, EMBRAIN (www.embrain.com) during the period between 10 and 18 January 2022. A total of 400 respondents were recruited from a pool of 1,650,000 public panels registered with the survey company. No specific exclusion criteria were applied. The estimated sample size was based on 40 samples for 10 cells (biological sex X five age groups) (20, 21). The survey company sent emails for enrollment emails to 2,000 ~ 3,000 panelists, and all 400 panelists’ responses were collected from 949 panelists who accessed the survey system.

The survey form reflected the guidelines (22) provided by the Checklist for Reporting the Results of Internet e-Surveys (CHERRIES). It included questions about participants’ demographic characteristics, responses to COVID-19, past psychiatric history, and current level of psychiatric distress as measured by symptom rating scales. Concerning COVID-19, participants were asked: “Did you experience quarantine because you had a COVID-19 infection?,” “Have you had a COVID-19 infection?,” and “Have you been vaccinated?.” Their past psychiatric history and current psychiatric status were assessed using their responses to the following questions: “Have you ever experienced or been treated for depression, anxiety, or insomnia?” and “Do you currently feel depressed, anxious, or in need of help to cope with your emotional state?.” The study protocol was approved by the Institutional Review Board (IRB) of the Asan Medical Center (2021–1755), which waived the need for written informed consent.

## Measures

### Questionnaires on adherence to physical distancing, health beliefs about COVID-19, and perceived social norms

We used Korean versions of the questionnaires translated in a previous study (23), using a translation/back-translation method. For each questionnaire, two bilingual experts separately translated the English version into Korean; these two Korean translations were merged into one, which was then back-translated into English by another bilingual expert. The back-translated and original English versions were compared to check for discrepancies in meaning, and the final Korean version was developed.

#### Adherence to physical distancing

We applied the physical distancing adherence questionnaire developed by Gouin et al. (18). Each of the seven items in this questionnaire (e.g., “I minimize contact with other people by staying at home”) can be rated on a 5-point Likert scale. Higher total scores indicate greater adherence.

#### Health beliefs about COVID-19 and perceived social norms

The questionnaire on COVID-19-related health beliefs included perceived susceptibility to infection (Three items, e.g., “How susceptible do you think you are to becoming infected or contracting the virus?”), perceived severity of viral infection (Three items, e.g., “If you become infected or contract the virus, how dangerous will COVID-19 be for you?”), the perceived benefits of physical distancing (Three items, e.g., “How effective do you think these social-distancing recommendations will be in protecting you from COVID-19?”), barriers to physical distancing (Four items, e.g., “How costly or expensive will it be for you to implement these recommendations?”), and self-efficacy (One item). To test the psychometric properties of the COVID-19 health beliefs questionnaire, the single self-efficacy item was not included, as it was the sole factor.

The questionnaire on perceived social norms related to physical distancing included one item each on descriptive social norms, personal injunctive norms or moral norms, and social injunctive norms, for a total of three items. Its psychometric properties were not investigated in this study because the three individual items could not be clustered into a single factor.

### Stress and anxiety to viral epidemics-6 items

The SAVE-6 is a 6-item scale that was developed to measure an individual’s viral anxiety (24); it is one of the subscales of the SAVE-9 scale, a self-report rating scale used to assess work-related stress and anxiety responses, specifically related to viral epidemics (25). Each item (e.g., “Are you afraid that your health will worsen because of the virus?”) can be rated using a 5-point Likert scale (0-never to 4-always). We used the Korean version of the SAVE-6 scale in this study. Cronbach’s alpha in this sample was 0.789.

### Patient health questionnaire-9

The PHQ-9 is a rating scale for assessing the severity of depression (26). It consists of 9 items, each of which (e.g., little interest or pleasure in doing things) can be rated on a 4-point Likert scale (0-not at all to 3-nearly every day). A higher total score corresponds to a higher level of depressive symptoms. We used the Korean version of the PHQ-9 (27), and Cronbach’s alpha was 0.890 in this sample.

### Statistical analysis

The construct validity and reliability of the Korean versions of the questionnaires on adherence to physical distancing and health beliefs were examined in the general population. The factor structure of both scales was examined through confirmatory factor analysis (CFA). For the CFA, the Kaiser-Meyer-Olkin (KMO) value and Bartlett’s sphericity test were used to examine sampling adequacy and data suitability. Next, the two-factor model for the Adherence to the physical distancing scale and the four-factor model for the Health Belief Model Scale were examined using the DWLS estimation. Model fit was assessed through a comparative fit index (CFI), Tucker-Lewis index (TLI), standardized root-mean-square residual (SRMR), and root-mean-square-error of approximation (RMSEA) values (28). Multigroup CFA was run to assess the measurement invariance of these two scales across gender, depression (PHQ-9 ≥ 10), and insomnia (ISI ≥ 8). The psychometric properties of these two scales were also examined using Rasch analysis. In the Rasch analysis, infit mean square (infit MnSq), outfit mean square (outfit MnSq), and item difficulty were assessed. Infit MnSqs and outfit MnSqs between 0.50 and 1.50 are recommended. In addition, item and person reliability and separation indices were calculated for both scales.

We also examined the interactions between the different assessments and the adherence to physical distancing. First, we performed correlation analysis using Pearson’s r. Then, based on the results, we constructed a structural equation model (SEM) in which the variables were arranged in such a way that the effects of each variable would ultimately lead to adherence to physical distancing.

The reliability of internal consistency was tested by Cronbach’s alpha and McDonald’s omega. Convergent validity was examined based on Pearson’s correlation analysis with scores on the SAVE-6 and PHQ-9 scales. We used SPSS version 21.0, AMOS version 27 for Windows (IBM Corp., Armonk, NY, United States), JASP version 0.14.1, jMetrik 4.1.1, and R version 4.1.2 with the lavaan package used to perform the statistical analyses.

## Results

### Reliability and validity of the questionnaires

Of the 400 participants, 204 (51.0%) were men, 52 (13.0%) had been quarantined, eight (2.0%) had been infected, and 368 (92.0%) had been vaccinated (Table 1). Before conducting the CFA, the suitability of the data and sampling was checked based on KMO measures (0.82 for both scales) and Bartlett’s test of sphericity (*p* < 0.001). Table 2 presents the factor loadings of the two-factor model of adherence to physical distancing and the four-factor model of health beliefs. CFA with DWLS estimation showed a good model fit for adherence to physical distancing (CFI = 1.000, TLI = 1.019, RMSEA = 0.000, and SRMR = 0.034) and health beliefs about COVID-19 (CFI = 0.993, TLI = 0.991, RMSEA = 0.030, and SRMR = 0.052, Table 3). The multi-group CFA showed that the Korean versions of the questionnaires on adherence to physical distancing and health beliefs about COVID-19 could be applied without considering gender, depression (PHQ-9 ≥ 10), or insomnia (ISI ≥ 8) (Supplementary Tables 1, 2).

**Table 1:** Clinical characteristics of the study subjects (*n* = 400).

Variable	Mean ± SD, *N* (%)
Male subjects, *n* (%)	204 (51.0%)
Age (years)	41.6 ± 10.8
18–29	86 (21.5%)
30–39	90 (22.5%)
40–49	108 (27.0%)
50–59	96 (24.0%)
≥ 60	20 (5.0%)
Marital status	
Single	186 (46.5%)
Married, with kids	169 (42.3%)
Married, no kids	35 (8.8%)
Other	10 (2.6%)
Questions about COVID-19	
Did you experience quarantine because you had a COVID-19 infection? (Yes)	52 (13.0%)
Have you had a COVID-19 infection? (Yes)	8 (2.0%)
Have you been vaccinated? (Yes)	368 (92.0%)
Psychiatric history	
Have you ever experienced or been treated for depression, anxiety, or insomnia? (Yes)	51 (12.8%)
Have you ever experienced or been treated for depression, anxiety, or insomnia? (Yes)	36 (9.0%)

**Table 2:** Factor structure of the Korean version of the physical distancing adherence and health beliefs questionnaires on COVID-19 and factor loadings (N = 400).

Items	Response scale					Descriptive		CITC	CID	Factor loading
	0	1	2	3	4	M	SD			CFA
(A) Questionnaire on adherence to physical distancing										
Distancing factor I										
Distancing 1	1.5	4.5	11.3	47.3	35.5	4.107	0.879	0.629	0.811	0.753
Distancing 2	0.8	4.0	13.3	41.5	40.5	4.170	0.859	0.664	0.806	0.802
Distancing 3	0.5	3.5	10.8	39.0	46.3	4.270	0.827	0.721	0.799	0.836
Distancing 4	1.5	4.0	11.3	35.8	47.5	4.238	0.910	0.569	0.819	0.650
Distancing 5	3.5	12.5	26.3	41.5	16.3	3.545	1.018	0.431	0.843	0.508
Distancing factor II										
Distancing 6	1.0	1.8	7.0	20.5	69.8	4.562	0.783	0.531	0.824	0.827
Distancing 7	0.3	1.3	6.3	20.3	72.0	4.625	0.682	0.512	0.826	0.717
(B) Questionnaire on health beliefs regarding COVID-19										
Perceived susceptibility										
Susceptibility 1	6.8	19.5	52.8	18.8	2.3	2.903	0.857	0.773	0.797	0.828
Susceptibility 2	4.0	18.5	56.3	19.5	1.8	2.965	0.781	0.809	0.767	0.877
Susceptibility 3	1.0	15.5	47.0	30.8	5.8	3.248	0.820	0.678	0.882	0.789
Perceived severity										
Severity 1	4.5	17.5	42.0	31.0	5.0	3.145	0.920	0.769	0.890	0.843
Severity 2	2.3	14.8	35.5	41.3	6.3	3.345	0.885	0.842	0.825	0.908
Severity 3	2.0	13.0	43.8	34.5	6.8	3.310	0.855	0.804	0.860	0.854
Perceived benefit										
Benefit 1	8.3	11.3	22.8	45.5	12.3	3.423	1.101	0.875	0.898	0.941
Benefit 2	5.8	11.8	21.8	38.3	22.5	3.600	1.128	0.851	0.917	0.869
Benefit 3	8.3	9.0	22.0	42.8	18.0	3.533	1.135	0.871	0.901	0.920
Perceived barrier										
Barrier 1	15.0	16.0	47.5	18.3	3.3	2.787	1.015	0.390	0.895	0.390
Barrier 2	8.8	16.5	30.0	31.5	13.3	3.240	1.143	0.778	0.734	0.894
Barrier 3	10.0	14.8	32.3	29.8	13.3	3.215	1.154	0.794	0.725	0.924
Barrier 4	15.5	22.3	38.0	20.0	4.3	2.752	1.074	0.717	0.765	0.777

CITC, Corrected Item-total Correlation; CID, Cronbach’s alpha if item deleted; CFA, Confirmatory Factor Analysis.

**Table 3:** Scale-level psychometric properties of the Korean version of the questionnaires on adherence to physical distancing and health beliefs regarding COVID-19.

Psychometric properties	Adherence to physical distancing		Health beliefs regarding COVID-19				Suggested cut-off
	Distancing Factor I	Distancing Factor II	Susceptibility	Severity	Benefit	Barrier	
Cronbach’s alpha	0.826	0.740	0.870	0.901	0.935	0.833	≥ 0.7
Standard error of measurement	1.443	0.668	0.790	0.765	0.996	0.914	Smaller than SD/2
KMO measure of sampling adequacy	0.82		0.82				0.5
Bartlett’s sphericity test	1052.00 (<0.001)		3660.844 (<0.001)				Significant
Confirmatory factor analysis model fits							
*χ*^2^ (df, *p* value)	5.456 (13, 0.964)		80.449 (59, 0.033)				Not significant
CFI	1.000		0.993				>0.95
TLI	1.019		0.991				>0.95
RMSEA	0.000		0.030				<0.08
SRMR	0.034		0.052				<0.08

KMO, Kaiser-Meyer-Olkin; CFI, comparative fit index; TLI, Tucker-Lewis index; RMSEA, root-mean-square-error of approximation; SRMR, standardized root-mean-square residual.

The physical distancing adherence questions showed good reliability for Factor I (Cronbach’s alpha = 0.826) and Factor II (Cronbach’s alpha = 0.740, Table 3). The four categories in the questionnaire on health beliefs about COVID-19 also showed good reliability (perceived susceptibility, Cronbach’s alpha = 0.870; perceived severity, Cronbach’s alpha = 0.901; perceived benefits, Cronbach’s alpha = 0.935; perceived barriers, Cronbach’s alpha = 0.833).

Rasch analysis results (Supplementary Table 3) showed that the infit and outfit MnSqs for both scales were within the recommended range (0.50 to 1.50). The item difficulty results showed that item 3 was the least difficult item and item 5 was the most difficult item in the questionnaire on adherence to physical distancing. For the COVID-19 health beliefs questionnaire, item 3 of the susceptibility to infection subscale was the least difficult item, and item 1 of the perceived severity of viral infection subscale was the most difficult. All subscales in both questionnaires had the acceptable item and person-separation indices and reliability, except for Factor II in the adherence to physical distancing scale. This would be due to fewer items (only two) of the subscale.

The convergent validity of each factor with each other and with rating scales of depression or viral anxiety are shown in Table 4.

**Table 4:** Correlation coefficients of each variable across all participants.

Variables	Age	1	2	3	4	5	6	7	8	9	10	11	12
1. Adherence to physical distancing, Factor I	0.16**												
2. Adherence to physical distancing, Factor II	0.09	0.41**											
3. Adherence to physical distancing, total	0.17**	0.96**	0.82**										
4. Perceived susceptibility	−0.06	0.10*	0.08	0.11*									
5. Perceived severity	0.01	0.12*	0.09	0.13*	0.62**								
6. Perceived benefit	0.26**	0.29**	0.17**	0.30**	0.26**	0.32**							
7. Perceived barrier	−0.10*	−0.07	−0.06	−0.08	0.14**	0.09	−0.28**						
8. Self-efficacy	0.08	0.25**	0.20**	0.27**	0.16**	0.19**	0.32**	−0.02					
9. Descriptive social norms	0.22**	0.14**	0.14**	0.16**	−0.03	−0.01	−0.03	−0.004	0.01				
10. Personal injunctive or moral norms	0.21**	0.33**	0.26**	0.35**	0.14**	0.20**	0.57**	−0.35**	0.32**	0.14**			
11. Social injunctive norms	−0.15**	−0.11**	−0.20*	−0.21**	−0.08	−0.09	−0.35**	0.27**	−0.22**	0.04	−0.36**		
12. SAVE-6	0.06	0.12*	−0.002	0.10*	0.48**	0.44**	0.25**	0.12*	0.13**	0.001	0.16**	−0.15**	
13. PHQ-9	−0.18**	0.05	−0.12*	0.005	0.12*	0.16**	0.02	0.26**	0.04	−0.16**	−0.13**	0.04	0.27**

SAVE-6, Stress and Anxiety to Viral Epidemics-6 items; PHQ-9, Patients Health Questionnaire-9 items *p < 0.05, **p < 0.01.

### Structural equation model

**Figure 1:**
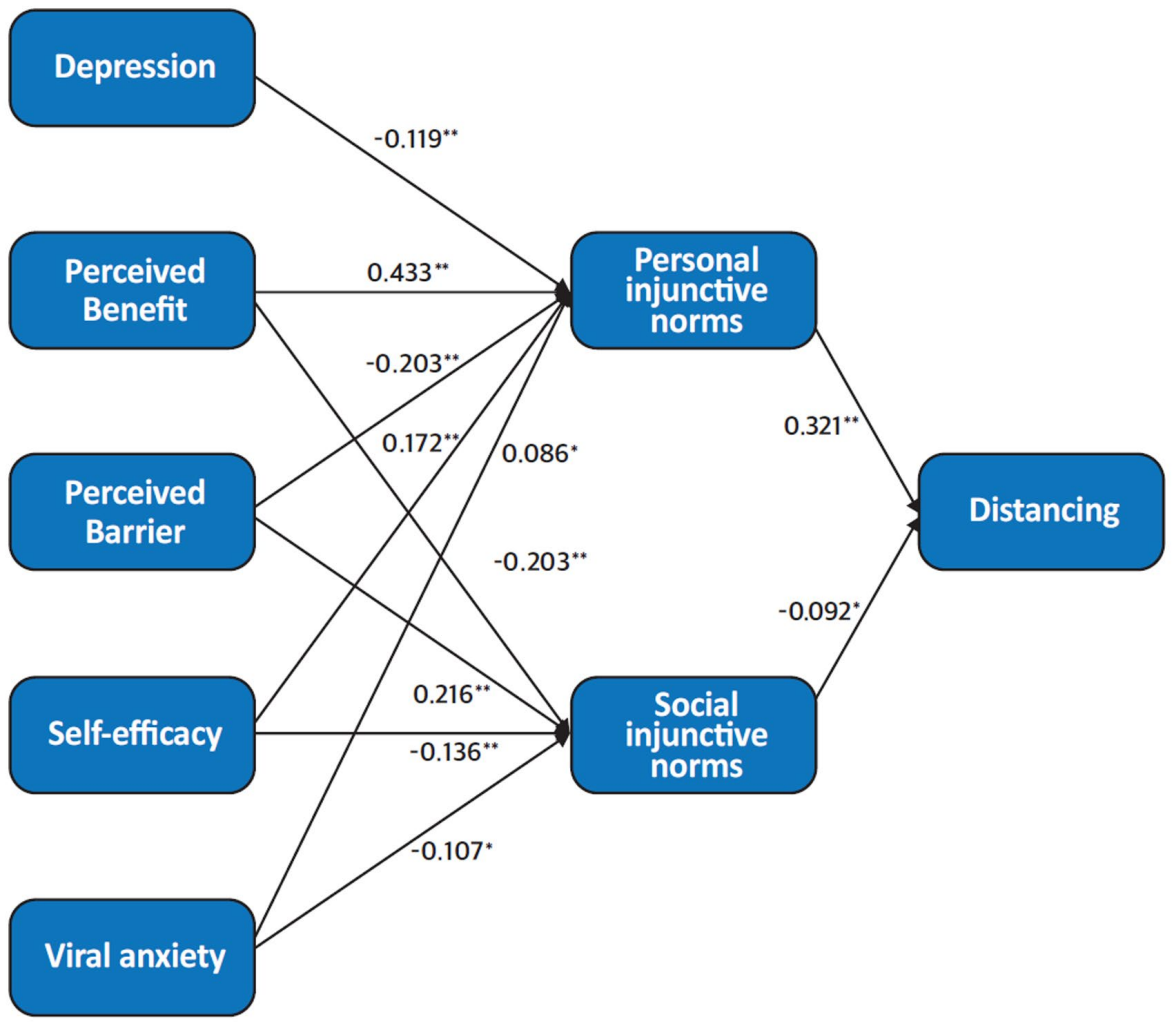
Structural equation model of the variables. Numbers next to arrows correspond to standardized estimates. The goodness-of-fit measures indicate a good fit (Chi-square = 24.425, df = 7, *p* < 0.001; CFI = 0.966; RMSEA = 0.073). †*p* < 0.1, *p < 0.05, **p < 0.01.

Based on the correlation results, we arranged the variables into three levels. The first level included viral anxiety, depression, perceived benefits, perceived barriers, and self-efficacy. The second level included personal injunctive norms and social injunctive norms. The final level consisted of adherence to physical distancing. The final model (Figure 1) showed that the effects of health beliefs and viral anxiety and depression were mostly mediated by personal injunctive norms. Goodness-of-fit measures indicated a good fit (Chi-square = 24.425, df = 7, *p* < 0.001; CFI = 0.966; RMSEA = 0.079).

## Discussion

This study aimed to validate the Korean versions of the questionnaires on physical distancing adherence and COVID-19-related health beliefs in the general population. We found them to be valid and reliable rating scales, which also included good convergent validity with measures of viral anxiety and depression. Through structural equation modeling, we also showed that personal injunctive norms were an important mediator, linking the effects of health beliefs to viral anxiety and depression.

In a previous study, we found that the two questionnaires on physical distancing adherence and COVID-19-related health beliefs could be applied to healthcare workers (23). The present study shows that these questionnaires can also be used in the general population. The results revealed a few differences, reflecting the different impacts of the pandemic. The first item in the perceived barrier subscale highlighted a key difference between these two groups. In response to the question, “How costly or expensive will it be for you to implement these recommendations?,” nearly half (48.0%) of healthcare workers responded “Not at all.” In contrast, a similar proportion of members of the public (47.5%) said “Moderately,” and more than 20% responded “A lot” or “Extremely,” despite holding views slightly more on the severe side of the perceived severity subscale. Factor loading was also relatively low (0.390), possibly for economic reasons. In South Korea, although the pandemic was a significant cause of emotional distress among healthcare workers, it rarely led to economic hardship or loss of work. After an initial period of shortages, the supply of personal protective equipment increased enough to keep prices affordable. During the same period, many small businesses saw their income shrink dramatically, possibly because of the social distancing measures. Members of the public also had a slightly more favorable view of the distancing measures on the perceived benefits subscale. How effective do you think these social-distancing recommendations will be in protecting you from COVID-19?”, 60% of the members of the public responded “A lot” or “extremely”, compared with only 40% of health care workers. While these results suggest that the public messaging campaign was successful in promoting social distancing in the general population, healthcare workers may have felt less positive, because many contracted the virus while working (29) despite strict adherence to distancing guidelines (30). These differences suggest that a change in perspective may be needed when addressing issues in these populations in the future.

The Rasch analysis showed that item 5 in the questionnaire on physical distancing, “In public, outside the home, stand at least two meters away from other people,” was the most difficult guideline to follow. Factor loading was also relatively low (0.508). This survey was conducted in January 2022. By that time in Korea, a significant number of individuals in the general public had already become accustomed to COVID-19 policies (and had grown less vigilant) after two years of the pandemic. They had received vaccinations (31) and had resumed many of their pre-pandemic activities, such as using public transportation for commuting and shopping at department stores. Government guidelines had also been relaxed. Consequently, during the survey period, it was challenging for participants to consistently adhere to a strict two-meter distance from others in practical life.

The CFA also showed a good fit for the four-factor model of the questionnaire on health beliefs regarding COVID-19, in parallel with our previous study (23). The subscales showed good convergent validity with each other and with other rating scales. However, the perceived barrier subscale score was not significantly correlated with the scores on adherence to physical distancing, a finding that we also observed in healthcare workers (23). Based on the Health Belief Model (HBM), individuals consider the effectiveness of an action or intervention in relation to perceived costs, dangers, unpleasantness, discomfort, time required, and inconvenience (32) Therefore, if people perceive a recommended policy to be more effective and the barriers to compliance to be low, we can expect them to adhere and comply with the policy. In contrast, if individuals perceive an extremely high level of severity or benefit, they may decide to follow the policy despite significant barriers. The pandemic may have had this effect; similar messaging strategies may be effective in future pandemics.

In addition, our structural equation model adds weight to the importance of personal injunctive norms or “moral norms” in a pandemic. Existing literature has already determined that it is independently associated with adherence to physical distancing, along with other measures of health beliefs (18). Our model supports these findings and goes further by showing that the effects of health beliefs on adherence to physical distancing are mediated by personal injunctive norms. These findings suggest that authorities should emphasize civic duties when educating the public and formulating public policy. Prior to COVID-19, humanity had already experienced numerous pandemics, which are now becoming more frequent (33). Since the preventive effect of physical distancing has already been proven, the government plays an important role in increasing policy adherence during any pandemic. For instance, depending on the health-belief model applied, public relations can emphasize the effectiveness of physical distancing and the risk posed by infectious diseases, thus encouraging people to participate in the policy. In addition, developing a non-contact social system and compensating for the losses caused by physical distancing will make the practice more accessible and sustainable. According to this study, the health belief model is mediated as a personal injunctive norm. Therefore, a political perspective that embraces different sociocultural classes is essential. This study has several limitations. First, the survey was conducted in January 2022, two years after the onset of the pandemic. Although participants may have adjusted their views on the pandemic during this time, they will also have had a chance to reflect on the effects of social-distancing measures. Second, the sample size was relatively small, limited to 400 individuals. Despite being large enough to meet the primary research objective (validating questionnaires for use with members of the general public), we were unable to compare differences between populations. Future studies with larger samples may be able to achieve this, while also uncovering factors to consider when targeting specific populations.

## Conclusion

In conclusion, the Korean version of the questionnaires on adherence to physical distancing and health beliefs with COVID-19 showed good reliability and validity in the general population. We also observed that the effects of health beliefs, along with viral anxiety and depression, were mainly mediated by personal injunctive norms. Health policymakers and healthcare professionals can use these questionnaires to assess adherence to physical distancing and health beliefs among the general population during the current pandemic. Our results also suggest that public-messaging strategies focusing on perceived severity, benefits, and civic duties may help to improve adherence to health interventions during future pandemics.

## Data availability statement

The raw data supporting the conclusions of this article will be made available by the authors, without undue reservation.

## Ethics statement

The study protocol was approved by the Institutional Review Board (IRB) of the Asan Medical Center (2021–1755), and obtaining the written informed consent was waived by IRB. Written informed consent for participation was not required for this study in accordance with the national legislation and the institutional requirements.

## Author contributions

YH, SY, and SC: conceptualization. SC, MA, and EC: data curation. OA, HA, and SC: formal analysis. YH, OA, HA, EC, MA, and SY: methodology. YH, HA, EC, OA, MA, SY, and SC: writing—original draft and writing—review and editing. All authors contributed to the article and approved the submitted version.

## Conflict of interest

The authors declare that the research was conducted in the absence of any commercial or financial relationships that could be construed as a potential conflict of interest.

## Publisher’s note

All claims expressed in this article are solely those of the authors and do not necessarily represent those of their affiliated organizations, or those of the publisher, the editors and the reviewers. Any product that may be evaluated in this article, or claim that may be made by its manufacturer, is not guaranteed or endorsed by the publisher.
