# Supplementary material for: Psychometric properties of the Korean version of questionnaires on adherence to physical distancing and health beliefs about COVID-19 in the general population
Source: Front Psychiatry. 2023 Jul 6;14:1132169. doi: 10.3389/fpsyt.2023.1132169 (PMC10356985; doi:10.3389/fpsyt.2023.1132169)
Supplement: Supplementary file 1 [file Table_1.DOCX]

**Supplementary Table 1. Measurement Invariance of questionnaire on adherence to physical distancing**

| **Model** | **χ^2^** | **df** | **Δ χ^2^** | **Δdf** | **p** | **CFI** | **ΔCFI** | **RMSEA** | **ΔRMSEA** |
| --- | --- | --- | --- | --- | --- | --- | --- | --- | --- |
| **Sex (male ve. female)** | | | | | | | | | |
| **Configural** | 10.056 | 26 |  |  |  | 1.000 |  | .000 |  |
| **Metric** | 14.481 | 31 | 4.425 | 5 | 0.490 | 1.000 | 0 | .000 | 0 |
| **Scalar** | 16.454 | 36 | 1.973 | 5 | 0.853 | 1.000 | 0 | .000 | 0 |
| **Strict** | 21.668 | 43 | 5.214 | 7 | 0.634 | 1.000 | 0 | .000 | 0 |
| **Depression (PHQ-9 ≥ 10)** | | | | | | | | | |
| **Configural** | 8.514 | 26 |  |  |  | 1.000 |  | .000 |  |
| **Metric** | 9.695 | 31 | 1.181 | 5 | 0.947 | 1.000 | 0 | .000 | 0 |
| **Scalar** | 10.107 | 36 | 0.412 | 5 | 0.995 | 1.000 | 0 | .000 | 0 |
| **Strict** | 16.004 | 43 | 5.897 | 7 | 0.552 | 1.000 | 0 | .000 | 0 |
| **Insomnia (ISI ≥ 8)** | | | | | | | | | |
| **Configural** | 8.640 | 26 |  |  |  | 1.000 |  | .000 |  |
| **Metric** | 10.211 | 31 | 1.571 | 5 | 0.905 | 1.000 | 0 | .000 | 0 |
| **Scalar** | 11.706 | 36 | 1.495 | 5 | 0.914 | 1.000 | 0 | .000 | 0 |
| **Strict** | 14.413 | 43 | 2.707 | 7 | 0.911 | 1.000 | 0 | .000 | 0 |

**Supplementary Table 2. Measurement Invariance of questionnaire on health beliefs about COVID-19**

| **Model** | **χ^2^** | **df** | **Δ χ^2^** | **Δdf** | **p** | **CFI** | **ΔCFI** | **RMSEA** | **ΔRMSEA** |
| --- | --- | --- | --- | --- | --- | --- | --- | --- | --- |
| **Sex (male ve. female)** | | | | | | | | | |
| **Configural** | 102.737 | 118 |  |  |  | 1.000 |  | .000 |  |
| **Metric** | 108.445 | 127 | 5.708 | 9 | 0.769 | 1.000 | 0 | .000 | 0 |
| **Scalar** | 112.213 | 136 | 3.768 | 9 | 0.926 | 1.000 | 0 | .000 | 0 |
| **Strict** | 119.079 | 149 | 6.866 | 13 | 0.909 | 1.000 | 0 | .000 | 0 |
| **Depression (PHQ-9 ≥ 10)** | | | | | | | | | |
| **Configural** | 97.4 | 118 |  |  |  | 1.000 |  | .000 |  |
| **Metric** | 101.395 | 127 | 3.995 | 9 | 0.912 | 1.000 | 0 | .000 | 0 |
| **Scalar** | 119.995 | 136 | 18.6 | 9 | 0.029 | 1.000 | 0 | .000 | 0 |
| **Strict** | 124.062 | 149 | 4.067 | 13 | 0.990 | 1.000 | 0 | .000 | 0 |
| **Insomnia (ISI ≥ 8)** | | | | | | | | | |
| **Configural** | 100.825 | 118 |  |  |  | 1.000 |  | .000 |  |
| **Metric** | 110.736 | 127 | 9.911 | 9 | 0.358 | 1.000 | 0 | .000 | 0 |
| **Scalar** | 115.85 | 136 | 5.114 | 9 | 0.824 | 1.000 | 0 | .000 | 0 |
| **Strict** | 120.523 | 149 | 4.673 | 13 | 0.982 | 1.000 | 0 | .000 | 0 |

**Supplementary Table 3. Infit and outfit MnSQs, and item and person separation index and reliability**

| **Items** | **Infit MnSq** | **Outfit MnSq** | **Difficulty** | **Item** | | **Person** | |
| --- | --- | --- | --- | --- | --- | --- | --- |
|  |  |  |  | **Separation index** | **reliability** | **Separation index** | **reliability** |
| **Adherence to physical distancing factor I** | | | | | | | |
| **Distancing 1** | .85 | .82 | -.06 | 6.775 | .979 | 1.793 | .763 |
| **Distancing 2** | .75 | .71 | -.22 |  |  |  |  |
| **Distancing 3** | .78 | .71 | -.49 |  |  |  |  |
| **Distancing 4** | 1.27 | 1.23 | -.40 |  |  |  |  |
| **Distancing 5** | 1.43 | 1.49 | 1.18 |  |  |  |  |
| **Adherence to physical distancing factor I** | | | | | | | |
| **Distancing 6** | .98 | .97 | .14 | .000 | 1.146 | .000 | .568 |
| **Distancing 7** | 1.00 | .99 | -.14 |  |  |  |  |
| **Perceived susceptibility** | | | | | | | |
| **Susceptibility 1** | 1.20 | 1.10 | .64 | 3.105 | .906 | 2.189 | .827 |
| **Susceptibility 2** | .80 | .73 | -.41 |  |  |  |  |
| **Susceptibility 3** | 1.00 | .81 | -.23 |  |  |  |  |
| **Perceived severity** | | | | | | | |
| **Severity1** | 1.20 | 1.10 | .64 | 3.105 | .906 | 2.189 | .827 |
| **Severity 2** | .80 | .73 | -.41 |  |  |  |  |
| **Severity 3** | 1.00 | .81 | -.23 |  |  |  |  |
| **Perceived benefit** | | | | | | | |
| **Benefit 1** | .88 | .68 | .44 | 2.368 | .849 | 2.365 | .848 |
| **Benefit 2** | 1.11 | 1.06 | -.38 |  |  |  |  |
| **Benefit 3** | .97 | .90 | -.06 |  |  |  |  |
| **Perceived barrier** | | | | | | | |
| **Barrier 1** | 1.77 | 1.85 | .40 | 5.330 | .966 | 2.175 | .825 |
| **Barrier 2** | .74 | .74 | -.45 |  |  |  |  |
| **Barrier 3** | .71 | .71 | -.40 |  |  |  |  |
| **Barrier 4** | .80 | .81 | .46 |  |  |  |  |
